# ROBO2 signaling in lung development regulates SOX2/SOX9 balance, branching morphogenesis and is dysregulated in nitrofen-induced congenital diaphragmatic hernia

**DOI:** 10.1186/s12931-020-01568-w

**Published:** 2020-11-18

**Authors:** Ana N. Gonçalves, Jorge Correia-Pinto, Cristina Nogueira-Silva

**Affiliations:** 1 Life and Health Sciences Research Institute (ICVS), School of Medicine, University of Minho, Campus de Gualtar, 4710-057 Gualtar, Braga, Portugal; 2 ICVS/3B’s - PT Government Associate Laboratory, Braga/Guimarães, Portugal; 3 Department of Pediatric Surgery, Hospital de Braga, Braga, Portugal; 4 Department of Obstetrics and Gynecology, Hospital de Braga, Braga, Portugal

**Keywords:** Branching, CDH, Epithelial progenitors, ROBO, SOX

## Abstract

**Background:**

Characterized by abnormal lung growth or maturation, congenital diaphragmatic hernia (CDH) affects 1:3000 live births. Cellular studies report proximal (SOX2^+^) and distal (SOX9^+^) progenitor cells as key modulators of branching morphogenesis and epithelial differentiation, whereas transcriptome studies demonstrate ROBO/SLIT as potential therapeutic targets for diaphragm defect repair in CDH. In this study, we tested the hypothesis that (a) experimental-CDH could changes the expression profile of ROBO1, ROBO2, SOX2 and SOX9; and (b) ROBO1 or ROBO2 receptors are regulators of branching morphogenesis and SOX2/SOX9 balance.

**Methods:**

The expression profile for receptors and epithelial progenitor markers were assessed by Western blot and immunohistochemistry in a nitrofen-induced CDH rat model. Immunohistochemistry signals by pulmonary structure were also quantified from embryonic-to-saccular stages in normal and hypoplastic lungs. Ex vivo lung explant cultures were harvested at E13.5, cultures during 4 days and treated with increasing doses of recombinant rat ROBO1 or human ROBO2 Fc Chimera proteins for ROBO1 and ROBO2 inhibition, respectively. The lung explants were analyzed morphometrically and ROBO1, ROBO2, SOX2, SOX9, BMP4, and β-Catenin were quantified by Western blot.

**Results:**

Experimental-CDH induces distinct expression profiles by pulmonary structure and developmental stage for both receptors (ROBO1 and ROBO2) and epithelial progenitor markers (SOX2 and SOX9) that provide evidence of the impairment of proximodistal patterning in experimental-CDH. Ex vivo functional studies showed unchanged branching morphogenesis after ROBO1 inhibition; increased fetal lung growth after ROBO2 inhibition in a mechanism-dependent on SOX2 depletion and overexpression of SOX9, non-phospho β-Catenin, and BMP4.

**Conclusions:**

These studies provided evidence of receptors and epithelial progenitor cells which are severely affected by CDH-induction from embryonic-to-saccular stages and established the ROBO2 inhibition as promoter of branching morphogenesis through SOX2/SOX9 balance.

## Background

Respiratory function depends on proximal or conducting airways that allow the continuous passageway of air and the distal or respiratory airways, where the gas exchange takes place [1, 2]. Interestingly, a family of transcription factors, SRY-related high-mobility-group, HMG, box (SOX) proteins, that bind DNA in a sequence-specific manner defined the proximal and distal arrangement during fetal life. Indeed, the fetal lung development starts from a multipotent stem cell population (SOX9 positive) that forms the most, if not all, conducting airways in a mechanism dependent on Wntless (Wnt), bone morphogenetic protein (BMP), and fibroblast growth factor (FGF) signaling ([3–10], reviewed in [11]). In brief, FGF10 directly activates β-Catenin in the distal epithelial progenitors, and induces BMP4 and SOX9 expression, to maintain and keep cells from differentiating into the SOX2 conducting airway epithelium. As the epithelial tube grows towards the distal source of FGF10, progeny from the distal multipotent epithelial progenitor are left behind in the epithelial stalk and once they are out of FGF reach they lose SOX9, β-Catenin and BMP4, start expressing SOX2, and differentiate into conducting airway epithelium ([3–10], reviewed in [11]). Finally, it is the separate capacity of SOX2 versus SOX9 cells to be differentiated in bronchiolar and alveolar lineages, respectively, that allows the morphological distinction between conducting and respiratory airways at the end of canalicular stage and pulmonary maturation at saccular phase in preparation for the first breath at birth [3, 4, 12–14].

Neonatal respiratory failure is common in moderate-to-severe cases of congenital diaphragmatic hernia (CDH). CDH is defined by a diaphragmatic defect that allows the herniation of abdominal organs into the thorax and consequently lung hypoplasia, affecting 1 in 3000 live-births [15, 16]. These hypoplastic lungs, strictly associated with high rates of mortality and morbidity, demonstrate a reduced number of terminal buds at early developmental stages (embryonic/pseudoglandular); reduced surface areas; and a decrease in distal branching and alveoli (saccular/alveolar) that impaired the efficient gas exchanges [17, 18]. Molecularly, the study of the underlying mechanisms demonstrated stage-dependent dynamics for BMP, WNT, or FGF pathways in experimental-CDH ([19–23], reviewed in [24]) with unexplored SOX2 or SOX9 profiles.

Genome-wide and transcriptome studies in CDH versus normal lungs in humans and rodents described high priority genes and pathways, such as ROBO/SLIT, as potential therapeutic target for the diaphragmatic defect repair [25, 26]. *Roundabout* (*Robo*) genes encode cell-surface receptors that respond to their secreted ligands, SLIT proteins, in a wide variety of cellular processes. In the lung, ROBO/SLIT knockout is inductor of diaphragm defect, delayed separation of foregut from the body wall [27], poor lung inflation and lethality at birth [27, 28]. On the other hand, ROBO/SLIT signaling is described in regulation of cell proliferation or progenitor cell profile in the development of mammary gland [29, 30], a branching organ-like lung, and in the central nervous system [31]. These intriguing functions as regulator of progenitor cell profile in development of mammary gland and central nervous system and inductor of the morphological defects in fetal lung development allowed us to hypothesize the ROBO1/2 as regulators of SOX2/SOX9 balance in fetal lung development, particularly in branching morphogenesis.

In this context, considering the intricate morphological, cellular and molecular dynamics during fetal lung development that determine the complex architecture of the lung at birth, the purpose of our investigation was determine the spatiotemporal distribution for receptors (ROBO1 and ROBO2) and epithelial progenitor markers (SOX2 and SOX9) from embryonic-to-saccular developmental stages in nitrofen-induced CDH rat model; and to identify the molecular effect of ROBO1 and ROBO2 inhibition in both ex vivo branching morphogenesis and SOX2/SOX9 profiles.

## Methods

### Animal model and experimental design

This study was carried out in strict accordance with FELASA guidelines [32] and with European (European Union Directive 86/609/EEC) regulation. The protocol was approved by the Direção Geral de Alimentação e Veterinária (DGAV 021328).

Female Sprague–Dawley rats (225 g; Charles-River; Spain) were maintained in appropriate cages under controlled conditions and fed with commercial solid food. The rats were mated and checked daily for vaginal plug. The day of plugging was defined as embryonic day (E) 0.5 for time dating purposes. According to the nitrofen-induced CDH rat model [33, 34], at E9.5, randomly selected pregnant rats were exposed to 100 mg of nitrofen (2,4-dichlorophenyl-*p*-nitrophenylether). At different time points (E13.5, E15.5, E17.5, E19.5, and E21.5), fetuses were harvested by cesarean section. After fetal decapitation, a thoracic-laparotomy was performed under a binocular surgical microscope (Leica, Wild M651.MSD, Switzerland) to inspect the diaphragm and harvest the organs.

The fetuses were divided into two groups: a control group (control) with fetuses exposed to olive oil alone, and hypoplastic group (hypoplastic) with those exposed to nitrofen. Regarding this experimental design, the assessment of diaphragmatic defects by surgical inspection at E13.5 and E15.5 is impractical. Therefore, for early gestational age, the hypoplastic group refers to the fetuses exposed to nitrofen (independently of CDH development), while at later gestational ages (E17.5, E19.5, and E21.5), the hypoplastic group refers to fetuses exposed to nitrofen which developed CDH. Lungs were either snap-frozen in liquid nitrogen for protein extraction or fixed in 4% paraformaldehyde for immunohistochemistry. Lungs of fetuses at 13.5 days post-conception were also dissected to perform fetal lung explants cultures and posterior Western blot analysis. GPower 3.1.9.4 (Franz Faul, Universitat Kiel, Germany) was used for sample size calculation. In total, 18 dams and 165 embryonic rats were used in this study.

### Immunohistochemistry

Normal and hypoplastic lungs of different gestational ages (E13.5–21.5) were fixed in 4% paraformaldehyde and embedded in paraffin as previously described [35]. 4 µm sections were placed onto glass microscope slides. Heat-induced antigen retrieval was performed with a citrate buffer. Sample sections were blocked with 4% fetal bovine serum for 1 h at room temperature and incubated with primary antibodies [ROBO1 (Cat No. sc25672) and ROBO2 (Cat No. sc16615), 1:200, overnight (ON), 4 ºC, Santa Cruz Biotechnology Inc, USA; SOX2 (Cat No. AF2018) and SOX9 (Cat No. AF3075), 1:100, ON, 4 ºC, R&D systems, USA] diluted in phosphate-buffered saline (PBS1x). Negative control reactions included omission of the primary antibody for which the immunoreactive staining was not observed. Tissue sections for negative control, ROBO1, SOX2, and SOX9 were labeled with a streptavidin–biotin immunoenzymatic antigen detection system (Cat No. TL-125-QHD, Thermo Scientific, USA) according to the manufacturer’s instructions and visualized with a diaminobenzidine tetrahydrochloride solution (Cat No. TA-125-QHDX, Thermo Scientific, USA) [36]. Tissues sections for ROBO2 and respective negative control were labeled with biotinylated anti-goat IgG (Cat No. BA-9500, Vector Laboratories, UK) followed by streptavidin–horseradish peroxidase incubation and visualized with a diaminobenzidine tetrahydrochloride solution (DAB, Cat No. TA-125-QHDX, Thermo Scientific, USA). Sections with and without primary antibodies were simultaneously processed and analyzed. The time expended in DAB solution was variable according to the developmental stage, but equally matched between control and hypoplastic sections, allowing the quantification of immunohistochemical signals. The percentage of stained cells per microscopic field was scored in four independent areas per section (four sections per each experimental group) by two blinded observers. Scoring was as follows: 0, 0–1% cells/pulmonary structure; 1, 1–25% cells/pulmonary structure; 2, 25–50% cells/pulmonary structure; 3, 50–75% cells/pulmonary structure; 4, 75–100% cells/pulmonary structure in accordance with [37]. At least three independent experiments were performed for each antibody tested. In each experiment, a different set of slides comprising the whole range of gestational ages was used. Different and unrepeated animal samples were selected for each group (gestational age). Six different animals were examined for each group per studied antibody. All sections were scanned with Olympus BX61 Upright Microscope (Olympus corporation, Japan) and independently evaluated by two investigators.

### Western blot analysis

Normal and hypoplastic lungs from different gestational ages (E13.5–E21.5) were processed for Western blot analysis in accordance with previously described methods [38, 39]. Briefly, 15 µg of protein were loaded onto 10% acrylamide mini gels, electrophoresed at 100 V at room temperature, and then transferred to nitrocellulose membranes (HybondTM-C Extra, GE Healthcare Life Sciences, UK). Blots were blocked in 5% bovine serum albumin and probed with primary antibodies to ROBO1 (1:500, ON, 4 ºC; Cat No. sc25672, Santa Cruz Biotechnology Inc., USA), ROBO2 (1:250, ON, 4 ºC; Cat No. sc16615, Santa Cruz Biotechnology Inc. USA), SOX2 (1:250, ON, 4 ºC; Cat No. AF2018, R&D system, USA), SOX9 (1:250, ON, 4 ºC; Cat No. AF3075, R&D system, USA), non-phospho (Active) β-Catenin (Ser33/37/Thr41) (1∶5000; Cat No. #4270, Cell Signaling Technology Inc., USA), total β-Catenin (1∶30,000; Cat No. #NBP1-54,467, NOVUS Biologicals, USA), and BMP4 (1:250, ON, 4 ºC; Cat No. sc-6896, Santa Cruz Biotechnology Inc., USA) according to the manufacturer's instructions. For loading control, blots were probed with β-Tubulin (1∶150.000; Cat No. ab15568, Abcam, USA). After this, membranes were incubated with a secondary horseradish peroxidase conjugate, developed with Clarity West ECL substrate (Cat No. 1705060, Bio-Rad, USA) and the chemiluminescent signal was captured using the Chemidoc XRS (Bio-Rad, USA) [36].

Quantitative analysis was performed with Quantity One 4.6.5 1-D Analysis Software (Bio-Rad, USA). Three independent experiments were performed (n = 3). In total, nine animals were used in each group (gestational age/condition) per antibody.

### Fetal lung explants

Harvesting and dissection of E13.5 lungs were made in PBS under a dissection microscope (Leica MZFLIII, Switzerland). The lungs were transferred to nucleopore membranes (Cat No. TETP01300, Whatman, USA), previously presoaked in Dulbecco's modified Eagle´s medium (DMEM) low glucose (Cat. No SH30021.01, Thermo Scientific, USA) for 1 h, and incubated in a 24-well culture plate (Nunc, Denmark). Floating cultures of the explants were incubated in a complete medium [50% DMEM, 50% nutrient mixture F-12 (Gibco, USA) supplemented with 100 µg/mL glutamine (Cat. No 25030081, Gibco, USA), 100 units/mL penicillin–streptomycin, (Cat. No 15140122, Gibco, USA), 0.25 mg/mL l-ascorbic acid (Cat No. A4403, Sigma-Aldrich, USA) and 10% fetal bovine serum (FBS) (Cat No. 26140079, Gibco, USA)].

The fetal lung explants were incubated in a 5% CO_2_ incubator at 37 °C for 96 h, and the medium was replaced every 48 h. The branching morphogenesis was monitored daily by photographing the explants. At day 0 (D0: 0 h) and day 4 (D4: 96 h) of culture, the total number of peripheral airway buds (branching) of all lung explants was determined by counting the number of peripheral airway epithelial buds of the developing respiratory tree [37, 40].

### ROBO1 and ROBO2 inhibitory studies

Ex vivo cultures of normal lung explants were complemented daily with distinct doses of recombinant rat ROBO1 Fc Chimera (0.04, 0.4, 4 and 40 ng/mL, R&D system; Cat No. 1749-RB-050), recombinant human ROBO2 Fc Chimera (0.04, 0.4, 4 and 40 ng/mL, R&D system; Cat No. 3147-RB-050), or recombinant human IgG1 Fc (used as control at higher dose, R&D; Cat No. 110-HG-100). These doses were selected according to the literature [41, 42]. Lung explants were obtained in three independent experiments (n ≥ 9 for each dose tested). After 4 days in culture, control, ROBO1 and ROBO2 inhibited lung explants were analyzed for branching morphogenesis in terms of area, external and internal epithelial perimeter, and the number of peripheral airway buds. In addition, SOX2, SOX9, BMP4, total and non-phospho (active) β-Catenin were quantified by Western blot at day 4 in all experimental groups.

### Statistical analysis

All quantitative data are presented as mean ± standard error of the mean (SEM). The statistical analysis was performed by two-way ANOVA for lung condition (normal and hypoplastic) and embryonic day (E13.5, E15.5, E17.5, E19.5, and E21.5) on protein expression level. One-way ANOVA was performed for the number of peripheral airway buds and protein expression levels on recombinant rat ROBO1 Fc Chimera (0.04, 0.4, 4, 40 ng/mL), and recombinant human ROBO2 Fc Chimera (0.04, 0.4, 4, 40 ng/mL). The parametric test assumptions were previously verified, and an additional LSD test was used for post-test analysis. Statistical analysis was performed using the statistical software IBM SPSS Statistics 24.0. Statistical significance was confirmed at p < 0.05.

## Results

### Experimental CDH alters the relative expression levels of ROBO1, ROBO2, SOX2, and SOX9 in fetal lung development

**Fig. 1:**
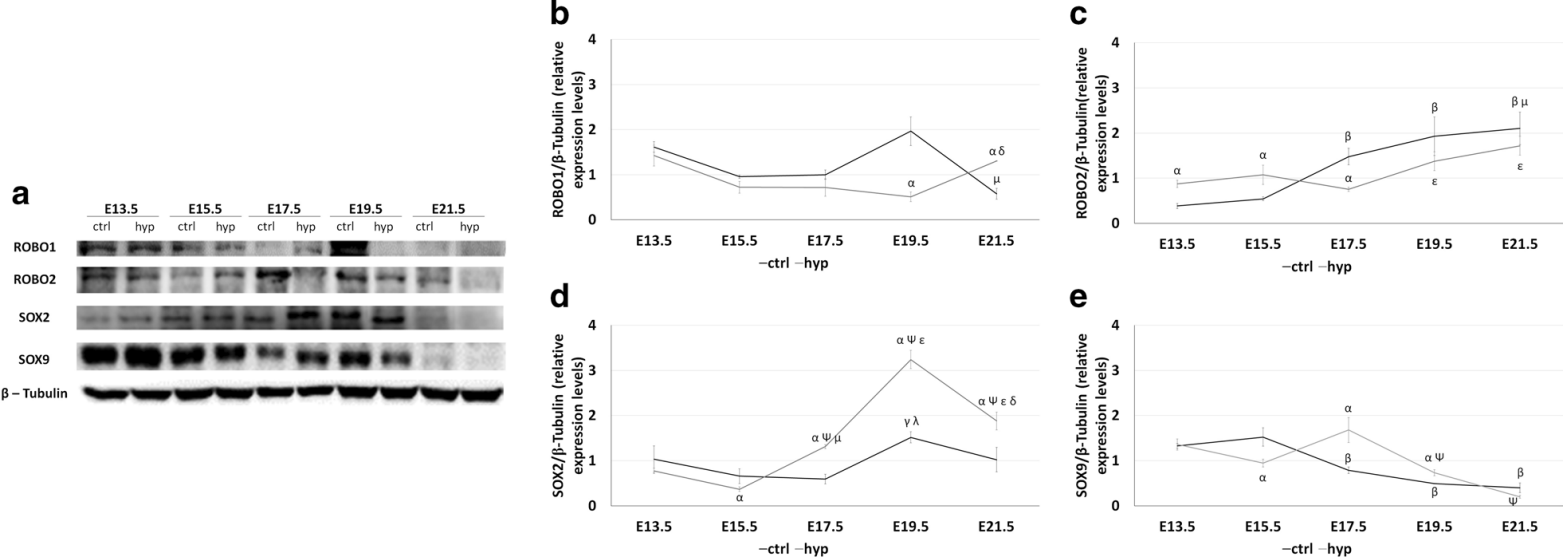
Distinct expression profiles for receptors and epithelial progenitors in normal and hypoplastic lungs. **a** Examples of representative blots are shown for each protein analyzed. Western blot analysis of **b** ROBO1; **c** ROBO2; **d** SOX2; and **e** SOX9 relative expression levels in normal (ctrl) and hypoplastic (hyp) lungs at selected gestational ages from E13.5-to-E21.5. Each lane represents a pooled-tissue sample, and relative expression was determined against β-Tubulin. Semi-quantitative analysis of three independent experiments for each protein is plotted (n ≥ 9 per timepoint and experimental groups, respectively). Results are presented as mean ± SEM. Symbols indicate the main effects and non-redundant interactions of the two-way ANOVA. p < 0.05: ^α^vs. ctrl; ^β^vs. E13.5- and 15.5-ctrl; ^Ψ^vs E13.5- and E15.5-hyp; ^γ^vs E15.5-ctrl; ^µ^vs E15.5-hyp; ^λ^vs E17.5-ctrl; ^ε^vs E17.5-hyp; ^δ^vs E19.5-hyp

To determine the molecular changes for ROBO1, ROBO2, SOX2 and SOX9 in experimental-CDH, the relative expression levels of each protein were analyzed by Western blot at the distinct gestational ages: E13.5, E15.5, E17.5, E19.5, and E21.5. Subsequent analysis revealed distinct molecular profiles for receptors (Fig. 1a–c) and epithelial progenitor markers (Fig. 1d and e), in normal and hypoplastic fetal lung development. Specifically, the progress of normal fetal lung development was defined by unchanged ROBO1 (Fig. 1b) and SOX2 (Fig. 1d) expression levels at E13.5, E15.5, E17.5 and E21.5, with E19.5 as the only affected stage in which the overexpression of ROBO1 (Fig. 1b) and SOX2 (Fig. 1d) was visible. In contrast, separate molecular profiles for ROBO1 (Fig. 1b) and SOX2 (Fig. 1d) were visualized in the experimental-CDH. While on the one hand, the ROBO1 (Fig. 1b) was decreased at E19.5 and overexpressed at E21.5, the SOX2 (Fig. 1d) was revealed to be downregulated at E15.5 and overexpressed at later gestational ages (E17.5-, E19.5-, and E21.5-hypoplastic versus control).

Regarding the relative expression levels of ROBO2 (Fig. 1c) and SOX9 (Fig. 1e), Western blot analysis identified an opposite effect on ROBO2 and SOX9 at later (E17.5, E19.5 and E21.5) versus early (E13.5, E15.5) developmental stages, in which the significant overexpression of ROBO2 and SOX9 depletion marked the normal lungs. In contrast, after experimental-CDH induction, ROBO2 (Fig. 1c) was significantly increased at E13.5 and E15.5 and downregulated at E17.5, while SOX9 (Fig. 1e) showed an important decrease at E15.5 and was overexpressed at E17.5 and E19.5 in experimental-CDH versus normal lungs.

To further explore these molecular profiles across hypoplastic versus normal fetal lung development, in vivo spatiotemporal distributions for ROBO1, ROBO2, SOX2, and SOX9 were analyzed by immunohistochemistry and quantified by pulmonary structure.

### Distinct ROBO1 and ROBO2 spatiotemporal dynamics during normal and hypoplastic fetal lung development

**Fig. 2:**
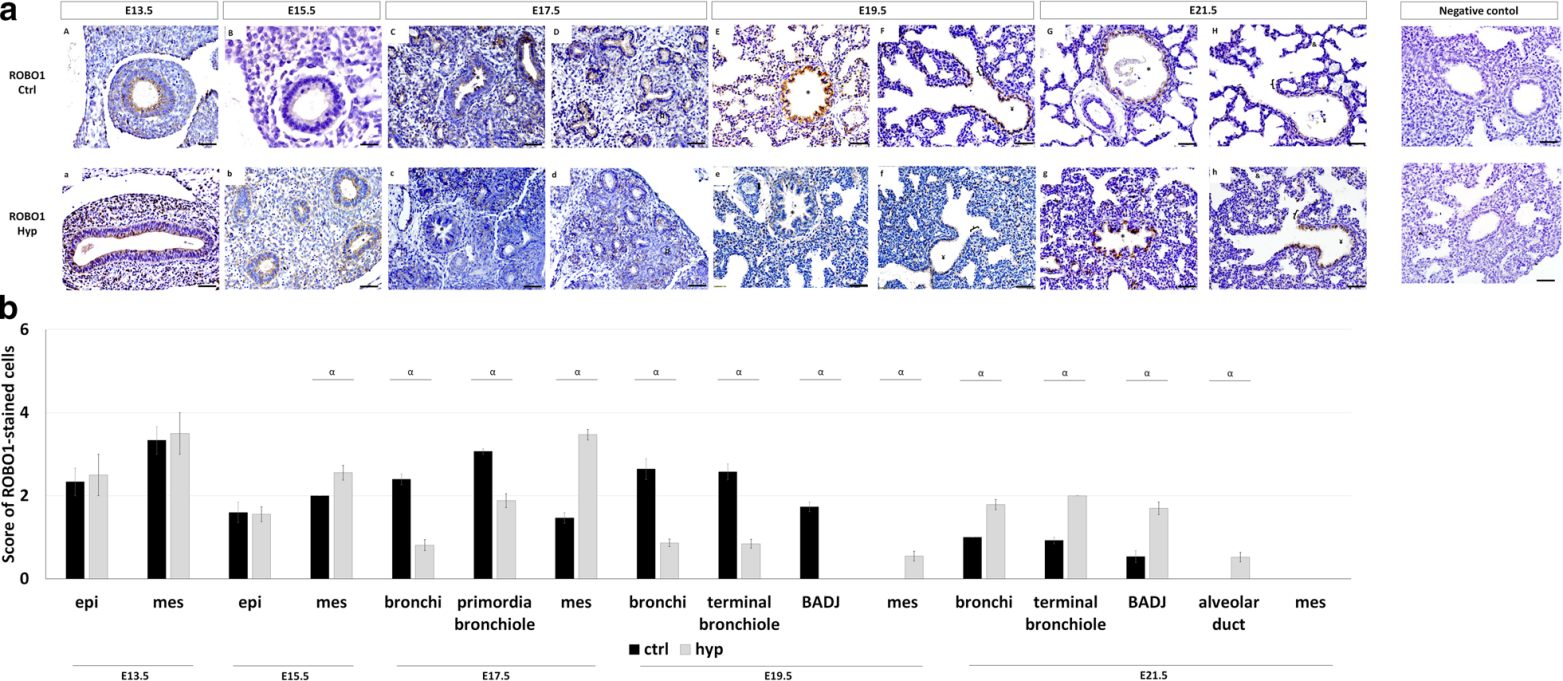
ROBO1 expression profile in normal and hypoplastic lungs. **a** representative immunohistochemical evidence for the presence of ROBO1 during normal (**aA–aH**) and hypoplastic (hyp, **aa–ah**) fetal lung development at the distinct gestational ages. Symbols in each figure identify the distinct pulmonary structures: *bronchi; ¤primordia of bronchiole; ¥terminal bronchiole; {bronchioalveolar duct junction; &alveolar duct. Data is representative of n ≥ 3 independent experiments per stage/protein. **b** ROBO1 stained cells quantified by pulmonary structure and developmental stage. Results are presented as mean ± SEM. Symbols indicate the main effects and non-redundant interactions of one-way ANOVA. ^α^p < 0.05. Scale bar 50 µm

Our results showed ROBO1 first expressed in mesenchymal and epithelial cells defining the undifferentiated tissues at E13.5 (Fig. 2aA) and E15.5 (Fig. 2aB). ROBO1+ was next expressed in bronchi and primordia of bronchiole (Fig. 2aC and aD) at E17.5, characterizing the first differentiated pulmonary structures in normal lungs. As lung development progressed, ROBO1 was observed in bronchi, terminal bronchiole, and bronchioalveolar junction (BADJ) at E19.5 (Fig. 2aE and aF) and E21.5 (Fig. 2aG and aH), with important gain-or-loss expression after experimental-CDH induction (Fig. 2aa–ah). Indeed, quantification of immunohistochemistry signals demonstrated the decrease of ROBO1 expression in bronchi at E17.5 and E19.5; primordia of bronchiole at E17.5; and terminal bronchiole at E19.5 (Fig. 2b). Conversely, the overexpression of ROBO1 was visualized in bronchi, terminal bronchiole, and BADJ at E21.5 in experimental-CDH versus normal lungs (Fig. 2b). Finally, unexpected new ROBO1+ cells were observed in the alveolar duct at E21.5 after induction of experimental-CDH (Figs. 2ag–ah and 2b).

**Fig. 3:**
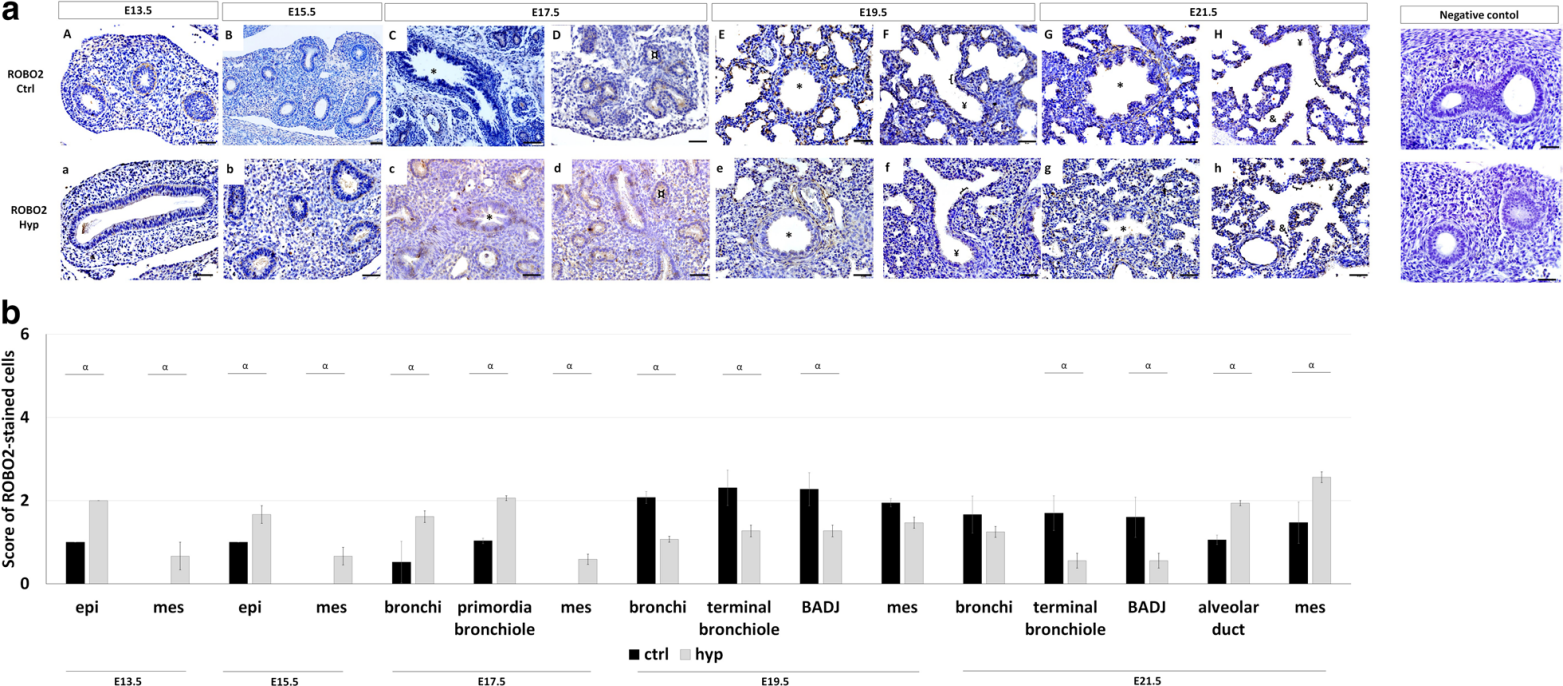
ROBO2 expression profile is altered after CDH-induction. **a** representative immunohistochemical assay for ROBO2 protein in normal (ctrl, **aA–aH**) and hypoplastic **(**hyp, **aa–ah**) lungs from 13.5-to-E21.5. Symbols in each figure identify the distinct pulmonary structures: *bronchi; ¤primordia of bronchiole; ¥terminal bronchiole; {bronchioalveolar duct junction; &alveolar duct. Data is representative of n ≥ 3 independent experiments per stage/protein. **b** ROBO2 expression levels by pulmonary structure and developmental stage. Results are presented as mean ± SEM. Symbols indicate the main effects and non-redundant interactions of one-way ANOVA. ^α^p < 0.05. Scale bar 50 µm

Regarding ROBO2, the immunohistochemistry assay demonstrated restricted epithelial ROBO2 expression in undifferentiated tissues, with ROBO2+ cells staining all pulmonary structures in normal (Fig. 3aA–aH) and hypoplastic (Fig. 3aa–ah) fetal lungs. Interestingly, compared to normal lungs, the quantification of stained cells indicated ROBO2 overexpressed in epithelium at E13.5 and E15.5; bronchi and primordia of bronchiole at E17.5; and in the alveolar duct and mesenchyme at E21.5 (Fig. 3b) in the nitrofen-induced CDH rat model. In contrast, a significant decrease in ROBO2 was observed in bronchi at E19.5; terminal bronchiole and BADJ at E19.5 and E21.5 (Fig. 3b) after experimental-CDH induction. New ROBO2+ cells were observed in mesenchyme at E13.5, E15.5, and E17.5 in experimental-CDH versus normal lungs.

### Damage of proximodistal patterning in experimental-CDH lungs

**Fig. 4:**
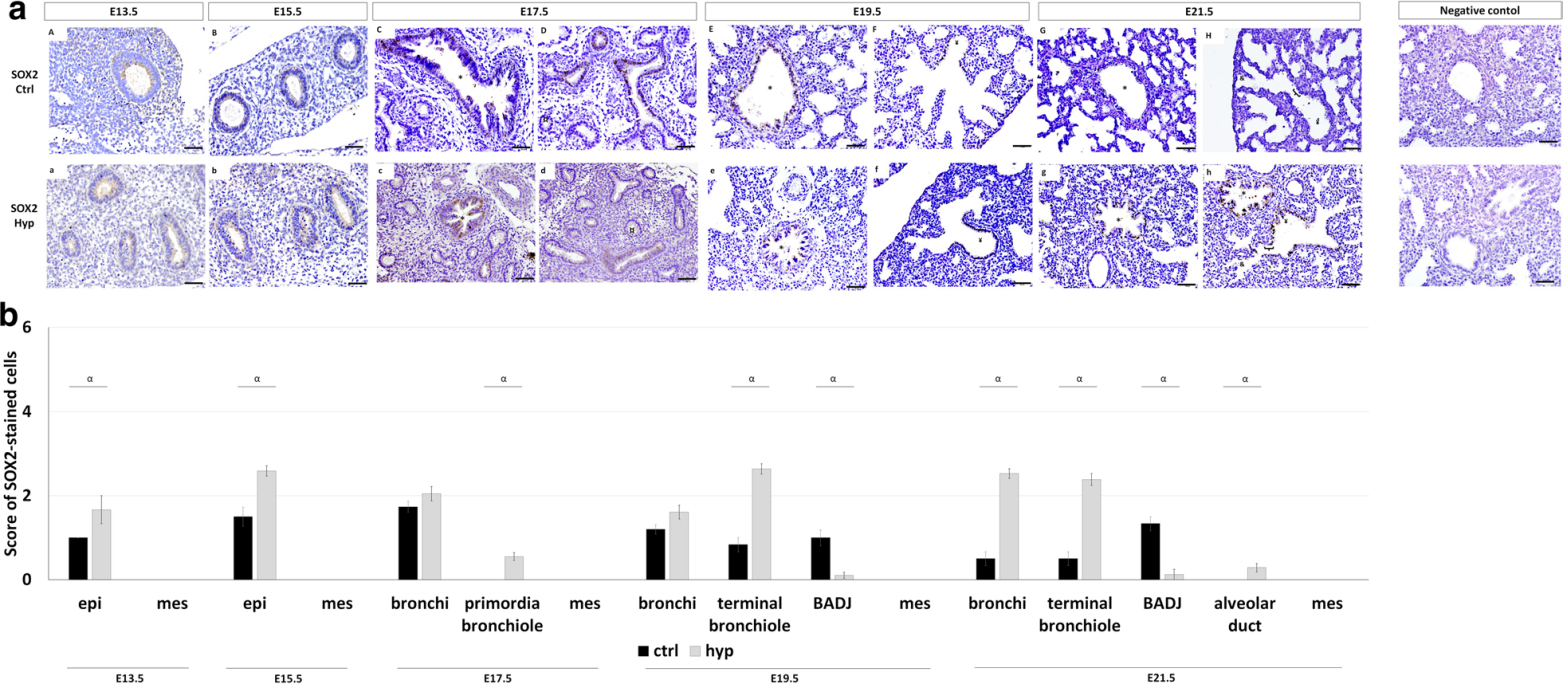
Experimental CDH alters SOX2 expression profile in fetal lung development. **a** representative immunohistochemistry at the distinct analyzed gestational ages in normal (ctrl, **aA–aH**) and hypoplastic (hyp, **aa–ah**) lungs. Symbols in each figure identify the distinct pulmonary structures: *bronchi; ¤primordia of bronchiole; ¥terminal bronchiole; {bronchioalveolar duct junction; &alveolar duct. Data is representative of n ≥ 3 independent experiments per stage/protein. **b** SOX2^+^ positive cells were quantified by pulmonary structure and developmental stage. Results are presented as mean ± SEM. Symbols indicate the main effects and non-redundant interactions of one-way ANOVA. ^α^p < 0.05. Scale bar 50 µm

To define the cellular profile of proximal and distal epithelial progenitors, the spatiotemporal distribution for SOX2 and SOX9, respectively, were analyzed across the distinct gestational ages (from E13.5 to E21.5) in normal and hypoplastic lungs. Immunohistochemistry revealed initial SOX2 (Fig. 4aA and aB; and Fig. 4aa and ab) expression in undifferentiated epithelial cells that progressively populate the proximal pulmonary structures in normal fetal lung development. Specifically, SOX2 was visualized in bronchi at E17.5 (Fig. 4aC and aD), E19.5 (Fig. 4aE and aF) and E21.5 (Fig. 4aG and aH); primordia of bronchiole at E17.5; terminal bronchiole and BADJ at E19.5 and E21.5 across normal fetal lung development. Regarding experimental-CDH, overexpression of SOX2 (Fig. 4b) was detected in the undifferentiated epithelium at E13.5 (Fig. 4aa) and E15.5 (Fig. 4ab); terminal bronchiole at E19.5 (Fig. 4ae and af) and E21.5 (Fig. 4ag and ah); and bronchi at E21.5. Interestingly, no SOX2+ cells were observed in primordia of bronchiole at E17.5 in experimental-CDH, whereas significant SOX2 loss characterized the BADJ at E19.5 and E21.5 (Fig. 4b).

**Fig. 5:**
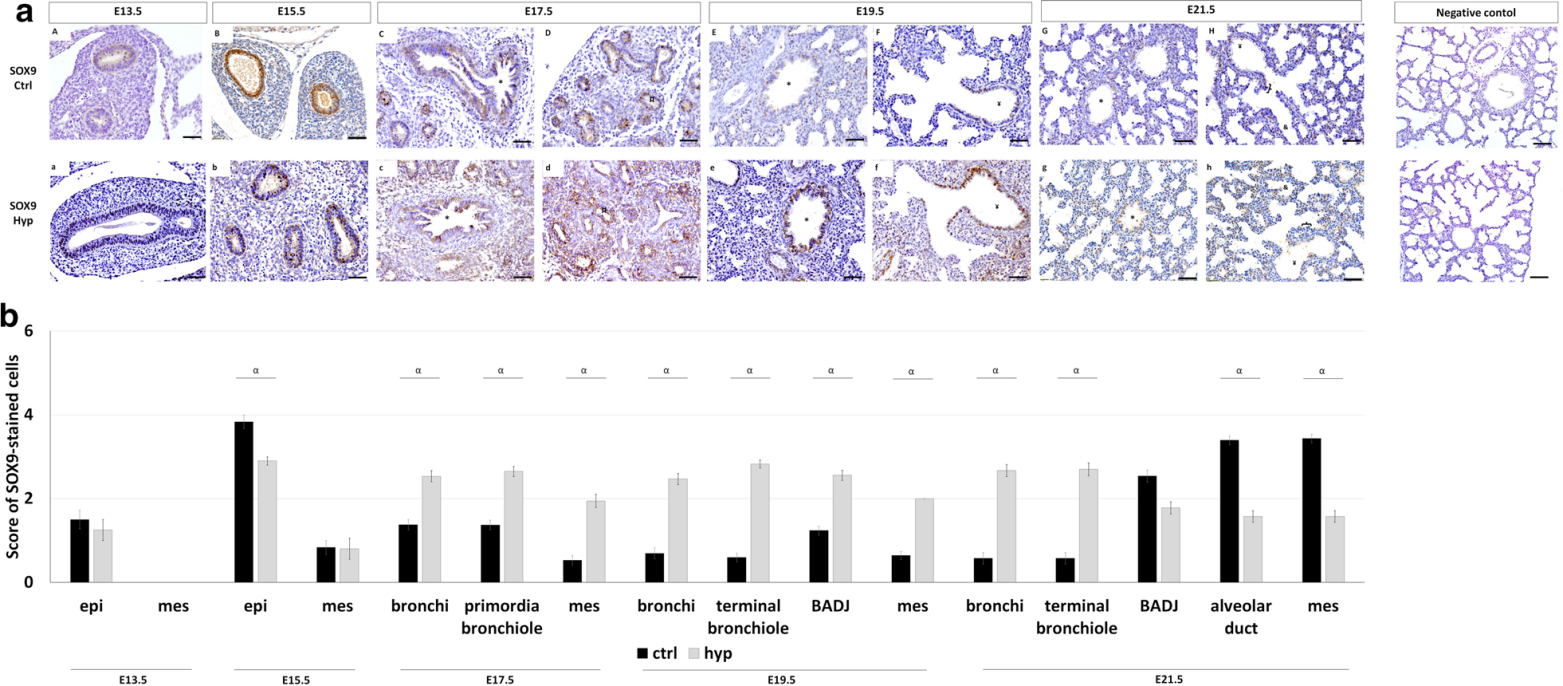
SOX9 spatiotemporal distribution in normal and hypoplastic lungs. **a** Representative immunohistochemical evidence SOX9 expression along the normal (ctrl, **aA–aH**); and hypoplastic (hyp, **aa–ah**) fetal lung development, from E13.5-to-E21.5. Symbols in each figure identify the distinct pulmonary structures: *bronchi; ¤primordia of bronchiole; ¥terminal bronchiole; {bronchioalveolar duct junction; &alveolar duct. Data is representative of n ≥ 3 independent experiments per stage/protein. **b** SOX9 stained cells quantified by pulmonary structure and developmental stage. Results are presented as mean ± SEM. Symbols indicate the main effects and non-redundant interactions of one-way ANOVA. ^α^p < 0.05. Scale bar 50 µm

Concerning SOX9, the immunohistochemistry assay identified an initial SOX9 expression in epithelium at E13.5 and E15.5 and mesenchyme at E15.5 that give rise to wide staining in all pulmonary structures in normal (Fig. 5aA–aH) and hypoplastic lungs (Fig. 5aa–ah). Indeed, SOX9 was detected in bronchi at E17.5, E19.5, and E21.5; primordia of bronchiole at E17.5; terminal bronchiole and BADJ at E19.5 and E21.5; and in alveolar duct at E21.5 in normal (Fig. 5aC–aH), and hypoplastic lungs (Fig. 5ac–ah) for which the more significant differences were observed after quantification of immunohistochemical signals (Fig. 5b). Particularly in experimental-CDH, SOX9 was downregulated in epithelium at E15.5 and in alveolar duct and mesenchyme at E21.5, whereas their overexpression was simultaneously observed in bronchi at E17.5, E19.5, and E21.5; primordia of bronchiole at E17.5; terminal bronchiole at E19.5 and E21.5; BADJ at E19.5; and mesenchyme at E17.5 and E19.5 (Fig. 5b).

These new findings urged us to explore the function of ROBO1 and ROBO2 in fetal lung development, particularly in branching morphogenesis and in SOX2/SOX9 expression profiles. For that, lung explant cultures at E13.5, previous reported as comparable to the in vivo lung both in structure and function, were selected as a model, in which restricted inhibition of ROBO1 or ROBO2 was performed followed by branching morphogenesis analysis and SOX2, SOX9, total and non-phospho (active) β-Catenin and BMP4 quantification at day 4.

### ROBO2 inhibition promotes branching morphogenesis

Ex vivo fetal lung explant cultures, at E13.5, were supplemented with IgG; recombinant ROBO1, or ROBO2 chimera proteins. When added to the culture medium, ROBO1 and ROBO2 chimera proteins selectively inhibit ROBO1 and ROBO2 function [41, 42]. In addition, apart from the ROBO1 and ROBO2 quantification, Western blot analysis was also performed for SOX2, SOX9, total, and non-phospho (active) β-Catenin and BMP4 at day4.

**Fig. 6:**
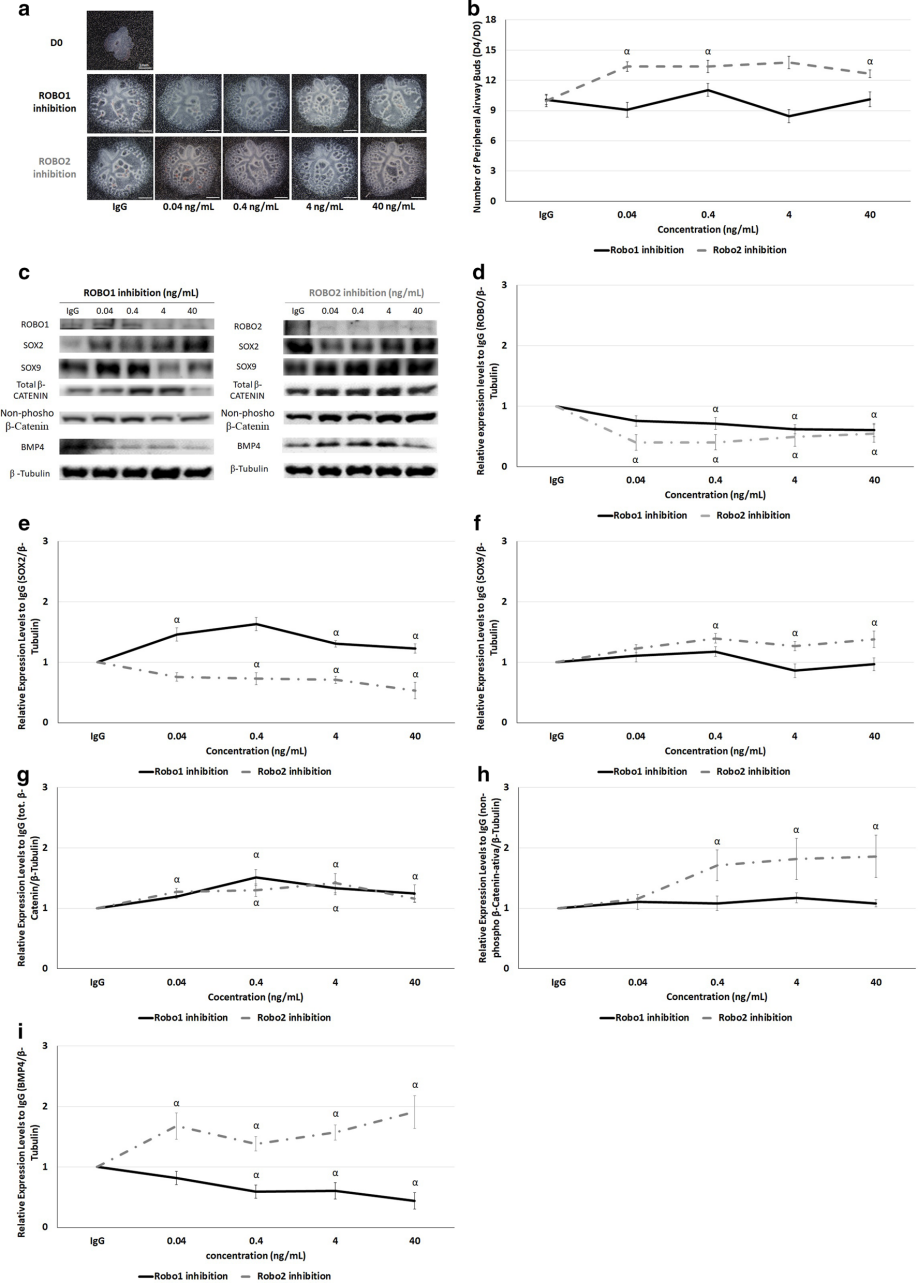
Effect of ROBO1 or ROBO2 functional impairment in branching morphogenesis. **a** The upper panel is representative of untreated lung explants (0 ng/mL) at D0; the bottom panel represents lung explants treated with recombinant IgG protein (0 ng/mL) and several doses of recombinant ROBO1 or ROBO2 proteins at day 4 (D4). **b** Morphometric analysis of the number of peripheral airway buds of fetal rat lung explants treated with increasing concentrations of recombinant ROBO1 (black) and ROBO2 (gray) proteins. Results are expressed as the D4/D0 ratio. **c** Examples of representative blots are showed for each analysed protein. Protein expression levels of **d** ROBO; **e** SOX2; **f** SOX9; **g** total β-Catenin; **h** non-phospho (active) β-Catenin; and **i** BMP4 in normal explant cultures treated with recombinant rat ROBO1 Fc Chimera for ROBO1 inhibition (black line) or recombinant human ROBO2 Fc Chimera for ROBO2 functional impairment (gray line). Recombinant human IgG Fc was used as control (IgG). n ≥ 9 per protein/condition. Each lane represents a pooled-tissue sample, and relative expression was determined against β-Tubulin and IgG. The data are presented as means ± SEM. p < 0.05: ^α^vs. IgG

Our results showed inhibition of ROBO2 as stimulator of branching morphogenesis from 0.04-to-40 ng/mL (Fig. 6a, b), while no significant differences in terms of number of peripheral airway buds were observed after ROBO1 inhibition (Fig. 6a, b). Molecular analysis showed significant ROBO1 and ROBO2 downregulation after supplementing the medium with recombinant rat ROBO1 Fc Chimera and recombinant human ROBO2 Fc Chimera, respectively, both versus recombinant human IgG Fc used as control (Fig. 6c, d). In addition, the overexpression of SOX2 (Fig. 6e) and total β-Catenin (Fig. 6g) with downregulation of BMP4 (Fig. 6i) and unchanged non-phospho (active) β-Catenin (Fig. 6h) and SOX9 (Fig. 6f) was identified after ROBO1 inhibition contrast, with a significative decrease in both SOX2 (Fig. 6e) and BMP4 (Fig. 6i); overexpression of SOX9 (Fig. 6f), non-phospho (active) (Fig. 6h) and total β-Catenin (Fig. 6g) observed after ROBO2 inhibition in promotion of fetal lung branching morphogenesis.

## Discussion

Despite the various distinct models used for the study of CDH, the nitrofen-induced CDH rat model is the most extensively used, particularly for the study of cellular and molecular mechanisms at early developmental stages since it produces diaphragmatic hernias and abnormal lungs. In fact, the nitrofen administration to pregnant dams midgestation causes developmental anomalies that reasonably replicate the major abnormalities and the pathophysiology described in human CDH, namely the specific location and extent of the diaphragmatic defects, lung hypoplasia, pulmonary hypertension, cardiovascular and skeletal defects (reviewed in [24], reviewed in [43]). On the other hand, the surgical model is employed to test promising antenatal therapies since it better mimics the later human developmental stage, alveolarization, and the cardiopulmonary transition at birth (reviewed in [24], reviewed in [43]). Our research aimed to determine the cellular/molecular alterations in CDH context at early developmental stages, embryonic-to-saccular, and thus the nitrofen-induced CDH rat model was the selected strategy.

**Fig. 7:**
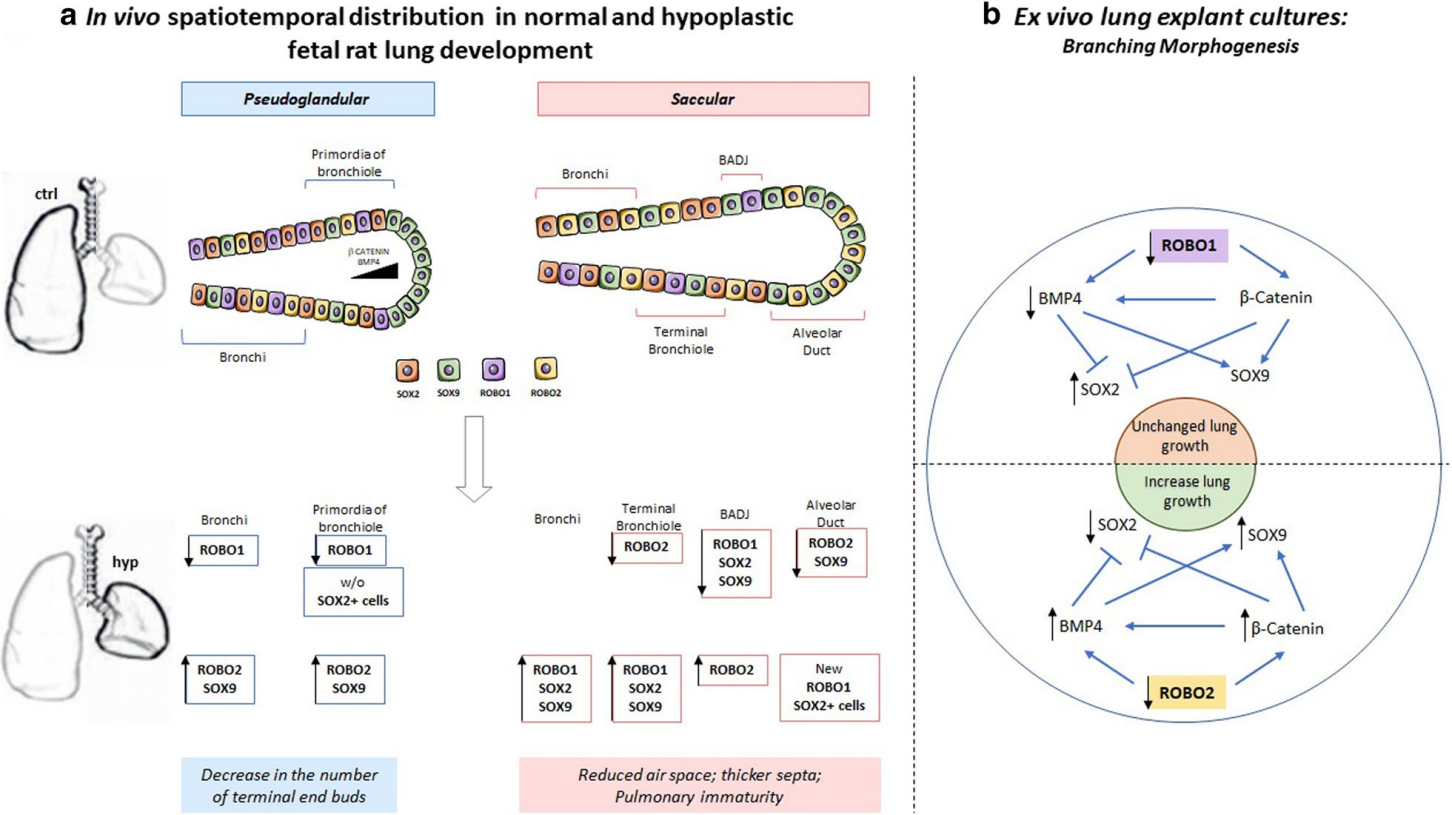
**a** Overview of the main changes in spatiotemporal distribution of ROBO1, ROBO2, SOX2 and SOX9 at pseudoglandular (E17.5) and saccular stages (E21.5) in hypoplastic (hyp) fetal lungs (lower panel). w/o without **b** A proposed model of ROBO regulation of ex vivo branching morphogenesis through BMP4, β-Catenin, SOX2 and SOX9

Our findings demonstrated changes in spatiotemporal distribution and relative expression levels for both receptors (ROBO1 and ROBO2) and epithelial progenitor markers (SOX2 and SOX9) in experimental-CDH versus normal lungs, according to pulmonary structure and developmental stage. In brief, the overexpression of ROBO2 and SOX2 in epithelium and the new ROBO2+ cells in mesenchyme defined the undifferentiated tissues in CDH. In contrast, at the time the first pulmonary structures are formed, distinct molecular profiles for ROBO1, ROBO2, SOX2 and SOX9 were visualized in epithelial cells (Fig. 7a), evidencing distinct capacities to react to molecular and environment signals in a hypoplastic lung.

Morphologic defects, such as decreased number of terminal buds at early and reduced air space and thicker septa at later developmental stages, characterize a CDH lung in both human infant and animal models. Underlying these morphological anomalies, our in vivo data demonstrated the impairment of proximodistal patterning from pseudoglandular-to-saccular stages (Fig. 7a), contributing to a better understanding of CDH pathogenesis. Indeed, the previous studies showed the proximodistal patterning formed at pseudoglandular stage building the functional units, i.e. the conducting airways, and respiratory airways at canalicular and saccular phases in preparation for the first breath at birth [3, 4, 12–14]. In addition, severe defects in branching morphogenesis and epithelial cell differentiation were observed after SOX2 [4, 13] or SOX9 [3] knockout. Our results also exposed distinct expression profiles suggesting three independent zones (bronchi and terminal bronchiole; BADJ; and alveolar duct) in a hypoplastic lung, able to distinctly react to molecular or environmental signals at E21.5. This study is the first to present SOX2/SOX9 analysis by pulmonary structure. In the adjacent literature, the molecular regulators of SOX2/SOX9 profiles (WNT, FGF, or BMP) have been confusedly reported in a separate studies with undefined conclusions ([19–23], reviewed in [24]), because of the limited information that can be taken from the protein quantification in the whole lung. Indeed, when we are looking for formation of the conducting and respiratory airways defined by proximodistal patterns, molecular and cellular analysis by pulmonary structure could be relevant, particularly in pathologic scenarios, since distinct mechanisms can be at work.

Therefore, our investigation provides novel observations concerning the impairment of SOX2 versus SOX9 profiles and the morphological defects in experimental-CDH. In this context, research revealing the mechanisms underlying SOX2/SOX9 expression in fetal lung development is of paramount importance since it allows the manipulation of branching morphogenesis and later the formation of conducting and respiratory airways. Using ex vivo lung explants cultures, we detected the ROBO2 inhibition as stimulator of fetal lung growth through activation of β-Catenin and BMP4 that promote SOX9 instead of SOX2 profiles. In contrast, the decrease of BMP4 and SOX2 increase visible after ROBO1 inhibition determines unaffected branching morphogenesis (Fig. 7b). Importantly, lung explant cultures are described as a useful method in the study of branching morphogenesis since they maintain the native physiological interaction between cells in the developing lung. Simultaneously, molecular analysis of the proximal and distal network in terms of SOX2, SOX9, BMP4 and active β-Catenin (target of Wnt pathway) (reviewed in [11]) allowed us to validate our ex vivo results regarding the molecular effects of ROBO2 inhibition in fetal lung growth and SOX9/SOX2 balance, since the proximodistal patterning is dependent on BMP4 and β-Catenin working in gradient in the distal tip of the lung (Fig. 7a).

Our in vivo observations also showed the overexpression of ROBO2 in lung hypoplasia, whereas the significant decrease of ROBO2 promotes the ex vivo branching morphogenesis, indicating a concordant role for ROBO2 in in vivo and ex vivo branching morphogenesis, respectively. In addition, there is similar inhibition of ROBO1 and overexpression of ROBO2 and SOX9 in bronchi and primordia of bronchiole with loss of SOX2+ cells only in primordia of bronchiole in experimental-CDH (Fig. 7a). This contrasts with the consistent opposite effect on SOX2 and SOX9 expression triggered by ROBO2 inhibition in ex vivo branching morphogenesis (Fig. 7b), providing evidence of the complexity of the in vivo model. Finally, genetic studies showing the impairment of SOX2 [44] and ROBO/SLIT signaling [25, 26] in human patients with CDH further validate the investigation of these targets in fetal lung development and particularly in CDH.

The literature has demonstrated a close relationship between epithelial and vascular development during fetal lung morphogenesis. Indeed, the downregulation of BMP, Wnt or transforming growth factor beta (TGFβ) signaling was previously associated not only with lung hypoplasia but also with pulmonary vascular remodeling, responsible for the development of persistent pulmonary hypertension at birth in a nitrofen-induced CDH rat model (reviewed in [21, 24, 45–48]). These observations are particularly relevant for in vivo studies since the development of the fetal lung and cardiovascular system cannot be separated when they divide the same molecular regulators, like BMP4. Our results showed that ROBO2 inhibition increases BMP4 expression and also branching morphogenesis. Thus, it is acceptable to suppose a simultaneous molecular effect of ROBO2 inhibition on epithelial and vascular branching, and consequent reversion of pulmonary hypoplasia and pulmonary hypertension in an experimental CDH context. In addition, the literature showed another SOX family member, SOX7, deleted in human infants with CDH [44] with functions in diaphragm formation [49], cardiovascular development [50] and in regulation of lineage decisions in cardiovascular progenitor cells [51] These functions are also identified for ROBO/SLIT signaling in fetal lung development, suggesting a potential association between SOX7 and ROBO/SLIT pathways.

Despite all the new findings reported here, we cannot ignore the limitations of our study regarding the in vivo applicability of this target and the challenge of treating a fetus patient, where several dysfunction pathways can be simultaneously targeted with one single treatment. Indeed, the careful analysis of our results suggests a limited applicability of ROBO2 in CDH. Although our observations report an upstream target that forms SOX9 instead of SOX2 progenitor cells at pseudoglandular stage, the literature showed distal and proximal progenitors giving rise to alveolar and bronchiolar lineages, respectively, in the next developmental stages in a mechanism dependent on both molecular and environment signals. In this context, the differentiated profile of the epithelial progenitor cells become unpredictable in a pathologic scenario, like CDH. Indeed, previous studies reported dysregulated factors involved in differentiation of airway epithelium in a nitrofen-induced CDH rat model (reviewed in [49]). Now, we describe cellular problems regarding the major coordinators of bronchiolar and alveolar differentiation, reinforcing the unanswered question: is the CDH pathogenesis a problem of differentiated or undifferentiated tissues?

## Conclusion

Our data provide the first evidence that receptors (ROBO1 and ROBO2) and epithelial progenitor markers (SOX2 and SOX9) are affected in experimental-CDH from embryonic-to-saccular stages and identified the ROBO2 inhibition as stimulator of branching morphogenesis and regulator of SOX2/SOX9 profiles through β-Catenin and BMP4 that could play important roles not only in stimulating in vivo lung growth, but also proximodistal patterning. Although ROBO2 inhibitors could be a promising therapeutic strategy for treatment of pulmonary hypoplasia related to CDH, more studies are necessary to carefully evaluate the differentiated capacity of these epithelial progenitor cells in the in vivo CDH context and the potential improvement in both lung morphological defects and neonatal respiratory function.

## Data Availability

Data sharing is not applicable to this article as no datasets were generated or analyzed during the current studies.

## Abbreviations

BADJ: Bronchioalveolar junction
BMP: Bone morphogenetic protein
CDH: Congenital diaphragmatic hernia
DAB: Diaminobenzidine tetrahydrochloride
DMEM: Dulbecco's modified Eagle medium
E: Embryonic day
FGF: Fibroblast growth factor
HMG: High-mobility-group
PBS: Phosphate-buffered saline
ROBO: Roundabout
SOX: SRY-related high-mobility-group box
Wnt: Wntless
